# Collaborative calculation and application of interreal and real interval relations to protect privacy

**DOI:** 10.1371/journal.pone.0261213

**Published:** 2021-12-14

**Authors:** Shaofeng Lu, Yuefeng Lu, Ying Sun

**Affiliations:** 1 School of Computer Science and Engineering, Northeastern University, Shenyang, China; 2 School of Civil and Architectural Engineering, Shandong University of Technology, Zibo, China; 3 State Key Laboratory of Resources and Environmental Information System, Institute of Geographical Sciences and Natural Resources Research, Chinese Academy of Sciences, Beijing, China; Victoria University, AUSTRALIA

## Abstract

The determination of the relation between a number and a numerical interval is one of the core problems in the scientific calculation of privacy protection. The calculation of the relationship between two numbers and a numerical interval to protect privacy is also the basic problem of collaborative computing. It is widely used in data queries, location search and other fields. At present, most of the solutions are still fundamentally limited to the integer level, and there are few solutions at the real number level. To solve these problems, this paper first uses Bernoulli inequality generalization and a monotonic function property to extend the solution to the real number level and designs two new protocols based on the homomorphic encryption scheme, which can not only protect the data privacy of both parties involved in the calculation, but also extend the number domain to real numbers. In addition, this paper designs a solution to the confidential cooperative determination problem between real numbers by using the sign function and homomorphism multiplication. Theoretical analysis shows that the proposed solution is safe and efficient. Finally, some extension applications based on this protocol are given.

## Introduction

With the rapid development of Internet technology, especially the rapid rise of big data computing, blockchains, artificial intelligence and other technologies, collaborative computing occupies an increasingly important position in humans’ daily work and learning. However, while the users are assisting in completing some computations, the need for the privacy and security of the data information of each cooperative participant is particularly urgent. Secure Multiparty Computation (SMC) was first proposed by A.C. Yao in 1982 in [1]. In [2], the theory of SMC has been further developed and laid its theoretical foundation. Secure multiparty computation mainly solves the problem that in a multiuser network in which users do not trust each other, each user can cooperate to perform a reliable computing task without disclosing their own private input information [3]. Therefore, secure multiparty computation has become a research hotspot in the field of cryptography in recent years [4] and is a core technology that can solve the collaborative computing problem to protect data information privacy.

In fact, it is impractical to use general protocols to solve some special instances in secure multiparty computation. In order to achieve high efficiency, some special methods are needed for some special problems [5]. In recent years, secure multiparty computation technology has been introduced by many scholars to the traditional fields of scientific computing, data mining, computational geometry, information retrieval and statistical analysis. Thus, new research directions, such as the correlation of protecting private information [6], privacy preserving cooperative scientific computations [7–9], Privacy Preserving Data Mining (PPDM) [10, 11], Privacy Preserving Computation Geometry (PPCG for short) [12–15], Private Information Retrieval (PIR) [16], Privacy Preserving Statistical Analysis (PPSA) [17], and the question of preserving the data ranking of private information [18–20], and secure multiparty quantum computation [21, 22] are generated, thus solving some important security application problems.

The relationship between and between numerical values is the core of the scientific computational problem of privacy protection. In reference [23], with the help of the theory of computational geometry, the input rational number or interval endpoint is taken as the straight line slope passing through the origin in the coordinate system, the problem of interval confidential calculation is transformed into the problem of a positional relation judgment between straight lines, and a solution providing a rational number and a rational interval confidential calculation is proposed. In reference [8], the positional relationship between rational numbers and rational intervals is transformed into other problems with the help of polynomials. It converts the problem into an integer vector to solve the issues of previous studies, which are confined to the rational number level and are still converted to an integer to solve. There are some limitations. The research purpose of this paper is to use the new technique to solve the decision problem of the relationship between the real and the real number interval and expand the real number level while improving the efficiency of the protocol. Furthermore, a new solution to the confidential cooperative determination problem between real numbers is designed by using the sign function and homomorphism multiplication.

### Contributions of this paper

First, by combining Bernoulli inequality generalization with a monotonic function, the scope of the data size comparison with privacy protection is extended to real numbers. In addition, before collaborative comparison calculation, by means of the Bernoulli inequality extension technique, the numerical range of real numbers to be compared is reduced to the interval level, and the addition and decryption operations are reduced. Based on the ElGamal encryption system and Paillier encryption system, this paper constructs a real number size comparison protocol.

Second, using symbolic functions and homomorphic encryption systems, an efficient cooperative decision protocol for confidentiality between real numbers is designed with certain techniques. Finally, the protocol is designed and applied to solve the problem of confidential data queries and the problem of confidential cooperative relationships between real numbers.

### Structure of this paper

Section 2 of this paper introduces the preparatory knowledge. In section 3, the designed protocol solves the problem of the collaborative calculation of the size comparison between real numbers to protect privacy. Section 4 designs a real number and the relationship between the real number confidentiality collaborative determination protocol. In section 5, the correctness and security of the protocol are analyzed, and simulation examples are used to prove that the protocol is secure. Section 6 analyzes the performance of the protocol, and Section 7 presents the specific extended applications of the three protocols. Section 8 summarizes this paper and forecasts future research directions.

## Preparatory knowledge

### Security definition

Semihonest participant [24]: In the secure multiparty computation protocol, participants are divided into three types according to their behaviors in the protocol: honest participants, semihonest participants and malicious participants. A semihonest participant in the implementation of the protocol will follow the protocol process in an honest way, but he may be corrupted by the attacker who discloses all his inputs, outputs and intermediate results to the attacker or deduces the information beyond the protocol or that of others based on the information he possesses.

Protocol security under the semihonesty model: According to Goldreich’s study [24], since the secure multiparty protocol under the semihonesty model can be transformed into the new protocol under the malicious model in most cases, this paper only designs the protocol under the semihonesty model and gives the corresponding security simulation examples.

Assume that the two parties involved in the calculation are Alice and Bob. Alice owns *x* and Bob owns *y*. They need to cooperate in the calculation of function *f*(*x*,*y*) = (*f*_1_(*x*,*y*),*f*_2_(*x*,*y*)) on the premise of ensuring the privacy of *x* and *y*. The purpose of the collaborative computation is that Alice and Bob obtain the two components of *f* and of *f*_1_(*x*,*y*) and *f*_2_(*x*,*y*), respectively. Let π represent the calculated protocol of *f*, and the information sequences obtained by Alice and Bob during the implementation of the protocol are respectively recorded as:

$$view_{1}^{\pi }(x,y)=(x,r_{1},m_{1}^{1},\cdots ,m_{1}^{s},f_{1}(x,y))\}$$ (1)

$$view_{2}^{\pi }(x,y)=(x,r_{2},m_{2}^{1},\cdots ,m_{2}^{t},f_{2}(x,y))\}$$ (2)

where *r*_1_ and *r*_2_ represent the independent random numbers of Alice and Bob, respectively; and $m_{1}^{i}(i=1\cdots ,s)$ represents the ith message received by Alice. After the execution of protocol π, the output of Alice is denoted as *f*_1_(*x*,*y*). $m_{2}^{j}(j=1\cdots ,t)$ represents the jth message received by Bob. After the execution of protocol π, Bob obtains the output, which is denoted as *f*_2_(*x*,*y*).

Definition: For protocol π that computes function *f*, the probabilistic polynomial time algorithms *S*_1_ and *S*_2_ are as follows:

$${\left\{S_{1}(x,f_{1}(x,y))\right\}}_{x,y}\overset{c}{\equiv }{\left\{view_{1}^{\pi }(x,y)\right\}}_{x,y}$$ (3)

$${\left\{S_{2}(y,f_{2}(x,y))\right\}}_{x,y}\overset{c}{\equiv }{\left\{view_{2}^{\pi }(x,y)\right\}}_{x,y}$$ (4)

Therefore, π computes the function *f* confidentially, where $\overset{c}{\equiv }$ is computationally indistinguishable.

Therefore, in the calculation of the two sides in the process of those protocols, only information from their input and output calculations can be obtained and the participants are unable to obtain the other party’s privacy information, thus proving that multiparty computation protocols are safe. You need to construct (3) and (4) set up the simulators of *S*_1_ and *S*_2_, respectively; therefore, the security method is called simulation examples.

### Paillier homomorphic encryption system

The Paillier encryption system is specifically described as follows [25].

Key generation: Select two large prime numbers *p* and *q* to calculate *N* = *pq* and *λ* = *lcm*(*p*−1,*q*−1). Define function $L(x)=\frac{x-1}{N}$ and randomly select a generator $g\in Z_{N^{2}}^{*}$ to make gcd(*L*(*g*^*λ*^ mod *N*^2^),*N*) = 1; then, the public key and private key of the encryption scheme are (*g*,*N*) and *λ*, respectively.

Encryption process: For plaintext *m*<*N*, random number *r*<*N* is selected to calculate ciphertext *c* = *E*(*m*).


$$c=g^{m}r^{N}\mathrm{mod}N^{2}$$ (5)

Decryption process: For ciphertext *c*, calculate plaintext *m* = *D*(*c*).


$$m=\frac{L(c^{\lambda }\mathrm{mod}N^{2})}{L(g^{\lambda }\mathrm{mod}N^{2})}\mathrm{mod}N$$ (6)

Additive homomorphism: Because the following property is true,

$$\begin{array}{l} D(E(m_{1})\times E(m_{2}))\\ =D(g^{m_{1}}r_{1}^{N}g^{m_{2}}r_{2}^{N}\mathrm{mod}N^{2})\\ =D(g^{m_{1}+m_{2}}{\left(r_{1}r_{2}\right)}^{N}\mathrm{mod}N^{2})\\ =(m_{1}+m_{2})\mathrm{mod}N, \end{array}$$ (7)

the Paillier encryption algorithm has additive homomorphism.

Homomorphic multiplication: Because the following property is true,

$$\begin{array}{l} D(E{\left(m_{1}\right)}^{m_{2}})\\ =D(g^{m_{1}m_{2}}r_{1}^{N}\mathrm{mod}N^{2})\\ =m_{1}m_{2}\mathrm{mod}N, \end{array}$$ (8)

the Paillier encryption algorithm has homomorphism multiplication.

### ElGamal homomorphic encryption system

The ElGamal encryption system is described as follows [26].

Key generation: Select parameter *k*, generate a large prime number *p* of *k* bits and a generator $g\in Z_{p}^{*}$, and randomly select $g\in Z_{p}^{*}$ as the private key; then, the corresponding public key is *h* = *g*^*x*^ mod *p*.

Encryption process: For plaintext $m\in Z_{p}^{*}$, random number *r* is selected to calculate the ciphertext.


$$c=E(m)=(c_{1},c_{2})=(g^{r}\mathrm{mod}p,mh^{r}\mathrm{mod}p)$$ (9)

Decryption process: For ciphertext c, calculate the plaintext *m* = *D*(*c*).


$$m=c_{2}\cdot c_{1}{}^{-x}\;\mathrm{mod}p$$ (10)

Multiplicative homomorphism: Since the following property is true,

$$\begin{array}{l} D(E(m_{1})\times E(m_{2}))\\ =D(\left(g^{r_{1}},m_{1}h^{r_{1}})\times (g^{r_{2}},m_{2}h^{r_{2}}\right))\\ =D(\left(g^{r_{1}+r_{2}},m_{1}\times m_{2}h^{r_{1}r_{2}}\right))\\ =(m_{1}\times m_{2})\mathrm{mod}p \end{array}$$ (11)

the ElGamal encryption algorithm has multiplicative homomorphism.

### Bernoulli inequality

The Bernoulli inequality is described as follows.

For any integer *n* and for any real number *h*, the following inequality holds:

$${\left(1+h\right)}^{n}\geq 1+nh,(n\in N^{*},h\in R,h>-1)$$ (12)

If *n* is positive and even, the Bernoulli inequality can be extended to any real number *h*∈*R*, that is:

$${\left(1+h\right)}^{n}\geq 1+nh,(h\in R,n\;\mathrm{is}\;\mathrm{positive}\;\mathrm{even}\;\mathrm{numbers})$$ (13)

Identification: When *h*>−1, the original inequality shows that for any integer *n*, (1+*h*)^*n*^≥1+*nh* is true.

When *h*≤−1, since *n* is even, (1+*h*)^*n*^≥0, 1+*nh*≤1−*n*<0, and (1+*h*)^*n*^≥1+*nh* are true.

Therefore, when *n* is even, the range of *h* in (1+*h*)^*n*^≥1+*nh* is any real number.

According to the promotion, we can derive the following two properties:

Property 1: For any real numbers *x* and *y*, *n* takes any even number. Then, if $y\geq {\left(1+\frac{x-1}{n}\right)}^{n}$, $y\geq {\left(1+\frac{x-1}{n}\right)}^{n}\geq 1+n\times \frac{x-1}{n}=x$, that is, *y*≥*x*.

Property 2: For any real numbers *x* and *y*, *n* takes any even number. Then, if $y\leq (1-n+nx^{\frac{1}{n}})$, $y\leq (1-n+nx^{\frac{1}{n}})\leq {\left(1+x^{\frac{1}{n}}-1\right)}^{n}=x$, that is, *y*≤*x*.

### Monotonic function

In general, let the domain of the function *f*(*x*) be *I*. Regarding the values of *x*_1_ and *x*_2_, for any two independent variables belonging to an interval of *I*, when *x*_1_>*x*_2_, *f*(*x*_1_)>*f*(*x*_2_), and then *f*(*x*) is an increasing function on this interval. When *x*_1_>*x*_2_, *f*(*x*_1_)<*f*(*x*_2_), and then *f*(*x*) is a negative function on this interval.

### Symbolic function

Set the function *sign*(*m*,*n*) as any two real variables *m* and *n* following the following rules:

$$sign(m,n)=\{\begin{array}{c} 1,m-n>0\\ 0,m-n=0\\ -1,m-n<0 \end{array}$$ (14)

The function is a symbol whose return value is the difference between the two real variables involved in the operation.

## Collaborative calculation of the size between real numbers to protect privacy

### Problem description and calculation principle

**Fig 1:**
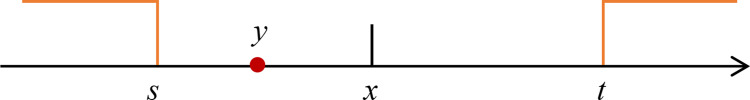
The relation between real numbers x and y.

Problem description: Alice has a real number *x* and Bob has a real number *y* (Fig 1). Through collaborative calculation, the two collaborative calculators can compare the size of the real number data they hold without revealing their own data information.

Calculation principle: For any two real numbers *x* and *y*, according to the generalization of the Bernoulli inequality above, for any even number *n*, if $y\geq t={\left(1+\frac{x-1}{n}\right)}^{n}$, then *y*≥*x*; and if $y\leq s=(1-n+nx^{\frac{1}{n}})$, then *y*≤*x*. In order to protect the data privacy, any positive even *n* value passed by the protocol in each round later in this paper is randomly generated. For the residual case, this paper uses a homomorphic encryption system to construct a monotonic function containing the residual interval.

### Specific protocol

Protocol 1. Collaborative calculation of the size between real numbers to protect privacy

Input: Alice has a real number *x*, and Bob has a real number *y*.

Output: The size comparison of *x* and *y* results in *sign*(*x*,*y*).

1. Alice sets a random positive even number *n* to construct $t={\left(1+\frac{x-1}{n}\right)}^{n}$. Then, Alice sends it to Bob.
2. Bob calculates *sign*(*t*,*y*), that is, Bob compares the size of *y* and *t* and returns the result *sign*(*t*,*y*) = -1. Then, the process ends. Otherwise, go to step 3.
3. Alice sets a random positive even number *n*′ to construct $s=(1-n^{\prime }+n^{\prime }x^{\frac{1}{n^{\prime }}})$ and then sends it to Bob.
4. Bob calculates *sign*(*s*,*y*), that is, Bob compares the sizes of *y* and *s* and returns the result *sign*(*s*,*y*) = 1. Then, the process ends. Otherwise, go to step 5.
5. Bob selects Paillier, a homomorphic encryption mechanism, to obtain (*g*,*N*) and *λ*. Then, he encrypts his data value *y* to obtain ciphertext *E*(*y*), *E*(*y*^2^) and sends it to Alice.
6. Alice selects random numbers *α*, *β* and *γ* to construct the monotonic function containing (*s*,*x*) in the defined domain, such as monotonic increasing function *f*(*x*) = *αx*^2^+*βx*+*γ* to calculate *f*(*s*) and *f*(*x*). At the same time, Alice performs a homomorphism operation on the received *E*(*y*), *E*(*y*^2^) to obtain ciphertext *c* = *E*(*y*^2^)^*α*^ *E*(*y*)^*β*^ *E*(*γ*) = *E*(*αy*^2^+*βy*+*γ*). Then, Alice sends *f*(*s*), *f*(*x*) and *c* to Bob.
7. After Bob decrypts the ciphertext *c* received, he gets *f*(*y*); and then he compares the sizes of *f*(*s*), *f*(*x*) and *f*(*y*). If *f*(*s*)≤*f*(*y*)≤*f*(*x*), output *sign*(*x*,*y*) = 1. Otherwise, output *sign*(*x*,*y*) = -1.

Protocol analysis: In the first four steps in the protocol, Alice constructs *x* into new values *t* and *s* by selecting random positive even numbers, and then these are sent to Bob. On his side, Bob simultaneously calculates the rules of the calculation result according to the symbol function and returns the value. Therefore, those involved in the simultaneous calculations of the two sides are unable to get any information on the other side. In the second part of the protocol, Bob’s master private key and his own *y* value encryption will be sent to Alice, so Bob’s information is not leaked to Alice. In addition, the monotonic function is constructed and mastered by Alice, and Bob cannot solve the monotonic function according to *f*(*s*) and *f*(*x*).

Next, we use the multiplicative homomorphism of the ElGamal encryption system to construct a similar protocol to solve the problem of the collaborative calculation of the size comparison between real numbers that protect privacy.

Protocol 2. Collaborative calculation of the sizes of real numbers to protect privacy

Input: Alice has a real *x*, and Bob has a real *y*.

Output: Size comparison of *x* and *y* results in *sign*(*x*,*y*).

1. Alice sets a random positive even number *n* to construct $t={\left(1+\frac{x-1}{n}\right)}^{n}$. Then, she sends it to Bob.
2. Bob calculates *sign*(*t*,*y*), that is, Bob compares the sizes of *y* and *t* and returns the result *sign*(*t*,*y*) = -1. Then, the process ends. Otherwise, go to step 3.
3. Alice sets a random positive even number *n*′ to construct $s=(1-n^{\prime }+n^{\prime }x^{\frac{1}{n^{\prime }}})$ and then sends it to Bob.
4. Bob calculates *sign*(*s*,*y*), that is, Bob compares the sizes of *y* and *s* and returns the result *sign*(*s*,*y*) = 1. Then, the process ends. Otherwise, go to step 5.
5. Bob selects ElGamal a homomorphic encryption mechanism, to obtain (*g*,*N*) and *λ*. Then, he encrypts his data value *y* to obtain ciphertext *E*(*y*), *E*(*y*^2^) and sends it to Alice.
6. Alice selects random numbers *α*, *β* and *γ* to construct the monotonic function *f*(*x*) containing (*s*,*x*) in the defined domain, the coefficients of function variables are required to be independent of the order of random numbers, to calculate *f*(*s*) and *f*(*x*). At the same time, Alice performs a homomorphism operation on the received *E*(*y*) to obtain ciphertext *c*. Then, Alice sends *f*(*s*), *f*(*x*) and *c* to Bob.
7. After Bob decrypts the ciphertext *c* received, he gets *f*(*y*), and then compares the sizes of *f*(*s*), *f*(*x*) and *f*(*y*). If *f*(*s*)≤*f*(*y*)≤*f*(*x*), *sign*(*x*,*y*) = 1 is output. Otherwise, *sign*(*x*,*y*) = -1 is output.

## Confidential collaborative determination of the interval relationship between real numbers

### Problem description and calculation principle

The problem description assumes that one of the two parties involved in the collaborative calculation has a real number and the other has an interval of real numbers. The two parties should work together to calculate the relationship between the real number and the interval of real numbers without revealing their respective data information. For example, Alice has a real number [*s*,*x*] and Bob has a real number interval *y*, and the two should secretly work together to determine the relationship between the real number and the interval of real numbers.

In order to determine whether a real number is in an interval, the calculation principle can determine whether a real number is in an interval by calculating the sign of the difference between the real number and the value at both ends of the interval, that is, it can determine whether a real number is in an interval of real numbers by using a sign function. For example, for a real number point *y*, to determine whether *y* is in an interval of [*s*,*x*], then the sign of *sign*(*y*,[*s*,*x*]) = *sign*((*x*−*y*)(*y*−*s*)) can be determined.


$$\begin{array}{l} sign(y,[s,x])\\ =sign(\left(x-y)(y-s\right))\\ =sign(y(x+s)-xs-y^{2}) \end{array}$$ (15)

From the above expansion, we can find the result value of *sign*(*y*(*x*+*s*)−*xs*−*y*^2^).

### Specific protocol

Protocol 3. Confidential collaborative determination of the interval relationship between real numbers

Input: Alice inputs real number [*s*,*x*], and Bob inputs real number interval *y*.

Output: Bob outputs *sign*(*y*,[*s*,*x*]).

The protocol constructed in this paper is as follows:

1. Alice selects Paillier, a homomorphic encryption mechanism, and obtains (*g*,*N*) for the public key and *λ* for the private key. Alice calculates *c*_1_ = −*xs* and *c*_2_ = *x*+*s* based on her data values *s* and *x*, encrypts *c*_1_ and *c*_2_, and obtains ciphertext *E*(*c*_1_) and *E*(*c*_2_). Then, she sends them to Bob.
2. Bob generates a random number *v*, encrypts the random number *E*(*v*). Then, Bob calculates *E*(*v*), *y* together with the received *E*(*c*_1_) and *E*(*c*_2_) as follows:
$$\bar{c}=E{\left(c_{2}\right)}^{y}\cdot E(c_{1})\cdot E(\nu )=E(yc_{2}+c_{1}+\nu ).$$
3. Bob sends $\bar{c}$ to Alice.
4. Alice decrypts $\bar{c}$ to obtain $\overset{⌢}{c}=y(x+s)-xs+\nu$ and sends $\overset{⌢}{c}$ to Bob.
5. Bob calculates:
$$\begin{array}{l} \hat{c}=sign(\overset{⌢}{c}-\nu -y^{2})\\ \;=sign(y(x+s)-xs-y^{2}) \end{array}$$
6. If $\hat{c}=1$, *y* is in the interval [*s*,*x*] or *y* is at the end point of the interval [*s*,*x*]; and $\hat{c}=-1$ means that *y* is outside the interval [*s*,*x*]. Then, Bob sends the result to Alice.

Protocol analysis: In Protocol 3, Alice’s data value *s* and *x* was mixed and encrypted before being sent to Bob who could not decrypt it. Bob added a random number *v* during the encryption operation, so although Alice decrypted $\bar{c}$, Alice could not obtain the specific information of *y* due to the existence of *v*.

## Correctness and safety analysis

### Correctness analysis

Protocol 1, in steps 1 to 4, according to the generalized properties 1 and 2 of Bernoulli inequality, the protocol can correctly calculate the size of the variables x and y and reduce the range of sizes to (*s*,*t*). In step 6, Alice chooses random numbers *α*, *β* and *γ*, the tectonic domain range contains the monotonic function of (*s*,*x*), and Alice calculates $c=E{\left(y^{2}\right)}^{\alpha }E{\left(y\right)}^{\beta }E(\gamma )=E(\alpha y^{2}+\beta y+\gamma )$. In step 7, Alice unlocks *c* and accesses *f*(*y*). According to the nature of the monotonic function, *s*, *x*, and *y* are consistent with *f*(*s*), *f*(*x*) and *f*(*y*), respectively; therefore, through comparing the sizes of *f*(*s*), *f*(*x*) and *f*(*y*), the function can be calculated within the scope of the (*s*,*t*) and *y* and the sizes of *s*, *x*, and *y*.

Similarly, the correctness analysis of Protocol 2 is similarly verifiable.

In Protocol 3, Bob calculates:

$$\begin{array}{l} \bar{c}=E{\left(c_{2}\right)}^{y}\cdot E(c_{1})\cdot E(v)\\ \;=E(yc_{2}+c_{1}+v)\\ \;=E(y(x+s)-xs+v) \end{array}$$

Alice decrypts to get ${\bar{c}}^{\prime }=y(x+s)-xs+\nu$. In step 4, Bob replaces *v* with −*y*^2^ to obtain *y*(*x*+*s*)−*xs*−*y*^2^ = (*x*−*y*)(*y*−*s*). Then, the specific sign value can be calculated according to the sign function. Therefore, protocol 3 is correct.

### Security analysis

Theorem 1. Protocol 1 can determine the size relationship between real numbers in a cooperative and confidential manner.

Proof: For the convenience of the description, this paper divides protocol 1 into two parts: part 1 is the first 4 steps, and part 2 is steps 5~7.

First, we will prove that the data are safe during the first 4 steps of the protocol’s execution.

In the first and third steps of the protocol, there are positive even numbers *n* and *n*′ randomly selected by Alice, and *t* and *s* are constructed. Because Bob does not obtain the values of *n* and *n*′, he cannot obtain the specific information for Alice.

In steps 2 and 4 of the protocol, Bob calculates according to information *t* and *s* his receives and his own data, and he only sends the symbol of the calculated result back to Alice, so Alice cannot obtain Bob’s specific information. Therefore, the first 4 steps of the protocol are safe.

The following simulation example is used to strictly prove the security of steps 5~7 of the protocol, that is, the simulators are constructed to make formulas (1) and (2) hold in the security definition.

In this protocol, let *f*_1_(*x*,*y*) = *f*_2_(*x*,*y*) = *sign*(*x*,*y*), and construct simulator *S*_1_. *S*_1_ accepts (*x*,*f*_1_(*x*,*y*)) as its input and proceeds as follows:

Step 5: Accept input (*x*,*f*_1_(*x*,*y*)) = (*x*,*sign*(*x*,*y*))

Since *S*_1_ has *sign*(*x*,*y*), it can pick any *y*′ that satisfies *sign*(*x*,*y*) = *sign*(*x*,*y*′).

*y*′ is encrypted according to protocol *S*_1_ to obtain ciphertext *E*(*y*′).

Step 6: *S*_1_ selects a random number *α*′, *β*′ and *γ*′ constructs the monotonic function containing (*s*,*x*) in the defined domain interval, and calculates *h*(*s*) and *h*(*x*). Simultaneously, it calculates ciphertext $c^{\prime }=E{\left({y^{\prime }}^{2}\right)}^{\alpha^{\prime }}E{\left(y^{\prime }\right)}^{\beta^{\prime }}E(\gamma^{\prime })=E(\alpha^{\prime }{y^{\prime }}^{2}+\beta^{\prime }y^{\prime }+\gamma^{\prime })$.

Step 7: After *S*_1_ decrypts ciphertext *c*′, it obtains *h*(*y*′) and calculates *sign*(*h*(*s*),*h*(*y*′)) and *sign*(*h*(*x*),*h*(*y*′)). If *sign*(*h*(*s*),*h*(*y*′)) = -1 and *sign*(*h*(*x*),*h*(*y*′)) = 1, output *sign*(*x*,*y*′) = 1. Otherwise, output *sign*(*x*,*y*′) = -1.

In this protocol,

$$view_{1}^{\pi }(x,y)=\{x,y,c,sign(x,y)\},$$

$$S_{1}(x,f_{1}(x,y^{\prime }))=\{x,y^{\prime },c^{\prime },sign(x,y^{\prime })\}.$$

Since *α*, *β*, *γ*, *α*′, *β*′ and *γ*′ are random numbers and *f*(*y*) = *αy*^2^+*βy*+*γ*, *h*(*y*′) = *α*′*y*′^2^+*β*′*y*′+*γ*′ and $f(y)\overset{c}{\equiv }h(y^{\prime })$ are derived.

Since *c* = *E*(*αy*^2^+*βy*+*γ*), *c*′ = *E*(*α*′*y*′^2^+*β*′*y*′+*γ*′), *α*, *β*, *γ*, *α*′, *β*′ and *γ*′ are random numbers and *c* and *c*′ are the same as the public key algorithm encryption results, *c* and *c*′ are indivisible, that is, $c\overset{c}{\equiv }c^{\prime }$.

Therefore, ${\left\{S_{1}(y,f_{1}(x,y))\right\}}_{x,y}\overset{c}{\equiv }{\left\{view_{1}^{\pi }(x,y)\right\}}_{x,y}$.

Similarly, simulator *S*_2_ can be constructed in a similar way so that:

$${\left\{S_{2}(y,f_{2}(x,y))\right\}}_{x,y}\overset{c}{\equiv }{\left\{view_{2}^{\pi }(x,y)\right\}}_{x,y}.$$

Therefore, protocol 1 can confidentially calculate the size relationship between real numbers.

Theorem 2. Protocol 2 can determine the size relationship between real numbers in a cooperative and confidential manner.

The proof of theorem 2 is similar to that of theorem 1 and will not be detailed in this article.

Theorem 3. Protocol 3 can determine the relationship between real points and intervals in a cooperative and confidential manner.

Identification: The security of the protocol is strictly proven by the simulation example below, that is, the emulators are constructed to make formula (1) and formula (2) hold in the security definition.

In this protocol, let *f*_1_(*y*,[*s*,*x*]) = *f*_2_(*y*,[*s*,*x*]) = *sign*(*y*,[*s*,*x*]) and construct the simulator *S*_1_. *S*_1_ accepts (*y*,*f*_1_(*y*,[*s*,*x*])) as the input and proceeds as follows:

Step 1: *S*_1_ accepts input (*y*,*f*_1_(*y*,[*s*,*x*])) = (*y*,*sign*(*y*,[*s*,*x*])). Since *S*_1_ has *sign*(*y*,[*s*,*x*]), it picks any [*s*′,*x*′] that satisfies *sign*(*y*,[*s*,*x*]) = *sign*(*y*,[*s*′,*x*′]). $c_{1}^{\prime }=-x^{\prime }s^{\prime }$ and $c_{2}^{\prime }=x^{\prime }+s^{\prime }$ are calculated according to protocol *S*_1_, and $c_{1}^{\prime }$ and $c_{2}^{\prime }$ are encrypted to obtain ciphertext $E(c_{1}^{\prime })$ and $E(c_{2}^{\prime })$, respectively.

Step 2: *S*_1_ generates a random number *v*′, encrypts the random number *E*(*v*′), and computes: ${\bar{c}}^{\prime }=E{\left(c_{2}^{\prime }\right)}^{y}\cdot E(c_{1}^{\prime })\cdot E(\nu^{\prime })=E(yc_{2}^{\prime }+c_{1}^{\prime }+\nu^{\prime })$.

Step 3: After *S*_1_ decrypts D, ${\overset{⌢}{c}}^{\prime }=y(x^{\prime }+s^{\prime })-x^{\prime }s^{\prime }+\nu^{\prime }$.

Step 4: Calculate $\begin{array}{l} {\hat{c}}^{\prime }=sign({\overset{⌢}{c}}^{\prime }-\nu^{\prime }-y^{2})\\ \;=sign(y(x^{\prime }+s^{\prime })-x^{\prime }s^{\prime }-y^{2}) \end{array}$.

In this protocol,

$$view_{1}^{\pi }(y,[x,s])=\{y,\bar{c},sign(y,[s,x])\},$$

$$S_{1}(y,f_{1}(y,[s,x]))=\{y,{\bar{c}}^{\prime },sign(y,[s^{\prime },x^{\prime }])\}.$$

Since *sign*(*y*,[*s*,*x*]) = *sign*((*x*−*y*(*y*−*s*)), *sign*(*y*,[*s*′,*x*′]) = *sign*((*x*′−*y*)(*y*−*s*′)), and

*sign*(*y*,[*s*,*x*]) = *sign*(*y*,[*s*′,*x*′]), *sign*((*x*−*y*)(*y*−*s*)) = *sign*((*x*′−*y*)(*y*−*s*′)).

Since $\bar{c}$ and ${\bar{c}}^{\prime }$ are the same as the public key algorithm encryption results, $\bar{c}$ and ${\bar{c}}^{\prime }$ are inseparable, namely, $\bar{c}\overset{c}{\equiv }{\bar{c}}^{\prime }$. Thus:${\left\{S_{1}(y,f_{1}(y,[s,x]))\right\}}_{y,s,x}\overset{c}{\equiv }{\left\{view_{1}^{\pi }(y,[x,s])\right\}}_{y,s,x}$ Similarly, simulator *S*_2_ can be constructed in a similar way so that: ${\left\{S_{2}(y,f_{2}(y,[s,x]))\right\}}_{y,s,x}\overset{c}{\equiv }{\left\{view_{2}^{\pi }(y,[x,s])\right\}}_{y,s,x}$.

Therefore, protocol 3 can confidentially calculate the relationship between real points and intervals.

## Efficiency analysis

This section analyzes the efficiency of Protocols 1, 2 and 3 and comparatively analyzes protocol 3 and protocol 1 in references [23] and [8] and protocol 3 in this paper. Because the protocol uses a homomorphic encryption mechanism, the number of costly modular exponentiation operations is taken as an indicator to measure the computing costs while the other operations are ignored. In the Paillier encryption scheme, one encryption or decryption requires two modular exponentiation operations. In the ElGamal encryption scheme, one encryption requires two modular exponentiation operations, and one decryption requires one modular exponentiation operation.

Computational complexity analysis and communication complexity analysis: Protocol 1 and Protocol 2 have negligible computational overheads in the first four steps. In the fifth to seventh steps of Protocols 1 and 2, both Alice operations need two modular exponentiation operations, and Bob performs encryption and decryption once each. Therefore, no more than six modular exponentiation operations are required in Protocol 1, and no more than five modular exponentiation operations are required in Protocol 2. Although protocol 1 and Protocol 2 need to perform 6 (or 5) modular exponentiation operations in the worst case, the protocol in this paper can complete the computations without requiring modular exponentiation operations in the best case.

Protocol 3 in reference [23] requires 13 modular exponentiation operations and 2 rounds of communication. Protocol 1 in reference [8] requires 12 modular exponentiation operations and 2 rounds of communication. In protocol 3 in this paper, Alice encrypts twice and decrypts once, which requires 6 modular exponentiation operations, Bob encrypts once and conducts ciphertext modular exponentiation operations once, which requires 3 modular exponentiation operations, a total of 9 modular exponentiation operations, and 2 rounds of communication.

In order to verify the efficiency of the protocol, we used the Java programming language to implement protocol 3 and the comparison protocol.

The experimental test environment is as follows: the operating system is the 64-bit Windows7 flagship version operating system, the processor is an Intel(R) Core(TM) i5-3470 3.20 GHz, and the amount of memory is 8.00 GB. The set value of each group was averaged after 1000 experiments. In the experiment, the large prime p and q bits used in the Paillier encryption algorithm are the same, both of which are 256 bits.

As seen in Tables 1 and 2, protocol 3 in this paper has fewer scheme modular exponentiation operations and has higher efficiency. Both the theoretical analysis and experimental results show that the protocol in this paper is efficient.

**Table 1:** Comparative analysis of the performance of the three protocols.

Protocol	Computational complexity	Communication complexity
Document [23] Protocol 3	13	2
Document [8] Protocol 1	12	2
Protocol 3 in this article	9	2

**Table 2:** Comparative analysis of the experimental results of the three protocols.

	Property		
	Alice’s running time (ms)	Bob’s running time (ms)	Total running time (ms)
Document [23] Protocol 3	16.246	15.328	31.574
Document [8] Protocol 1	15.412	12.831	28.243
Protocol 3 in this article	11.587	10.095	21.682

## Extended applications

### Confidential data query

The problem can be described as follows: one party participating in collaborative computing has an ordered data set, and the other party has a value to be searched. The two parties should cooperate to determine the specific location of the value in the data set without revealing their respective data information. For example, Alice has ordered data set {*c*_1_,*c*_2_,⋯*c*_*n*_}, Bob has data set *s*, and the two should work together confidentially to determine the specific location of numerical value *s* in data set {*c*_1_,*c*_2_,⋯*c*_*n*_}. Therefore, using binary search, protocol 1 or 2 can be used to compare the median of the data to be checked and the narrowing range of the data set for multiple times to achieve the required results.

### Cooperative determination of the relationship between real number intervals to protect privacy

**Fig 2:**
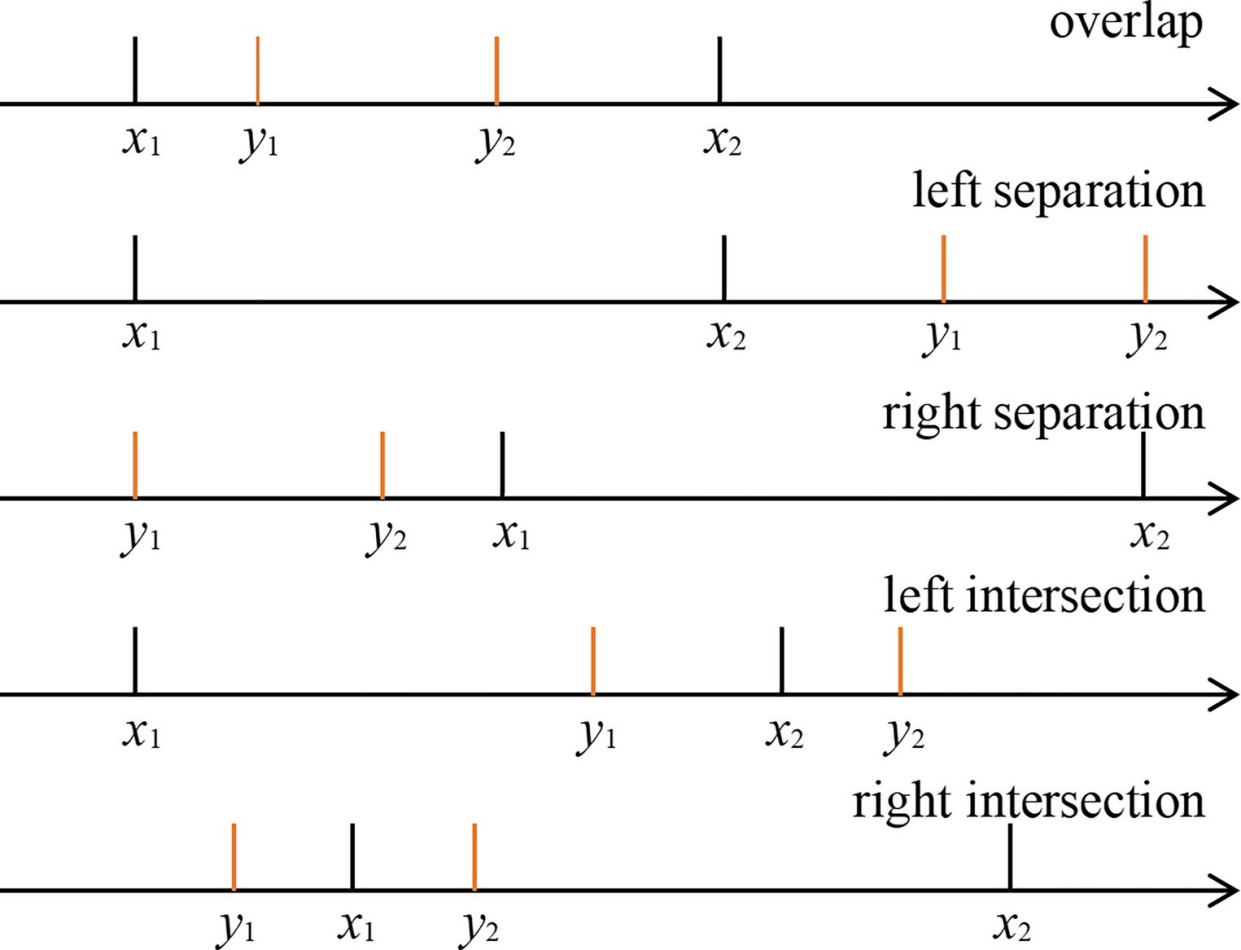
Five scenarios for |*x*_2_−*x*_1_|>|*y*_2_−*y*_1_|.

**Fig 3:**
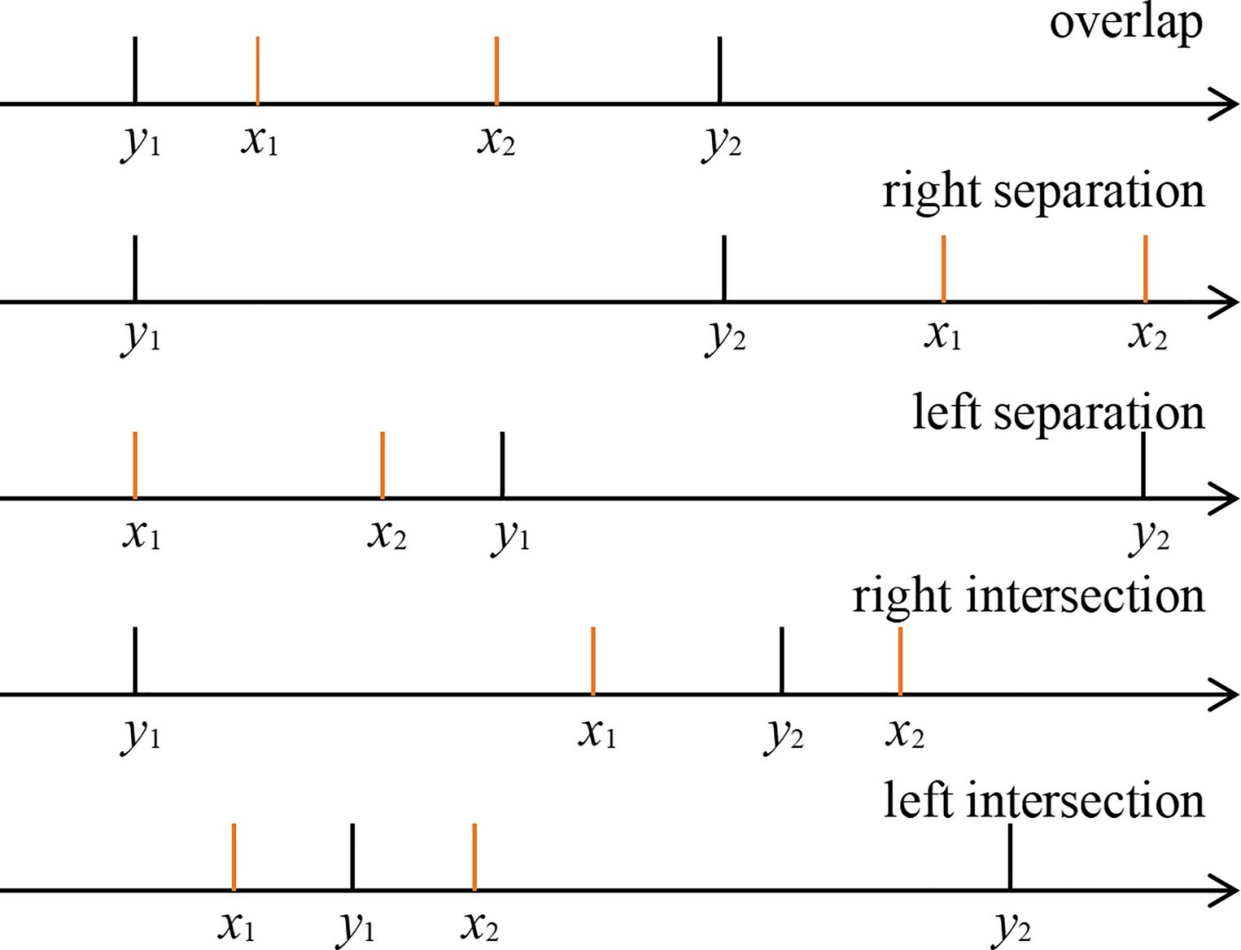
Five scenarios for |*x*_2_−*x*_1_|<|*y*_2_−*y*_1_|.

The problem can be described as follows: the two parties involved in the collaborative calculation have a real interval, and they should work together to calculate the relationship between the two intervals without revealing the numerical information of their respective intervals. For example, Alice has an interval [*x*_1_,*x*_2_], Bob has an interval [*y*_1_,*y*_2_], and they work together confidentially to determine the relationship between the two intervals. As shown in the Figs 2 and 3, the relationship between two real intervals can be divided into three categories: separation, intersection and overlap. This paper adopts the following methods to address this problem:

1. Alice calculates the magnitude of the interval [*x*_1_,*x*_2_] where *t* = |*x*_2_−*x*_1_|, and Bob calculates the magnitude of the interval [*y*_1_,*y*_2_] where *s* = |*y*_2_−*y*_1_|. Alice and Bob compare the sizes of *t* and *s*.
2. If *x*_2_<*y*_1_, then the left phase of the interval [*x*_1_,*x*_2_] is separated from the interval [*y*_1_,*y*_2_]; and if *x*_1_>*y*_2_, then the right phase of the interval [*x*_1_,*x*_2_] is separated from the interval [*y*_1_,*y*_2_].
3. When *t*<*s*, *x*_2_>*y*_1_>*x*_1_. If *y*_2_>*x*_2_>*y*_1_, then the left part of the interval [*x*_1_,*x*_2_] intersects at the interval [*y*_1_,*y*_2_]; and if *y*_2_>*x*_1_>*y*_1_, then the right part of the interval [*x*_1_,*x*_2_] intersects at the interval [*y*_1_,*y*_2_]. Otherwise, if *y*_1_,*y*_2_ is less than *x*_2_ and greater than *x*_1_, then the interval [*y*_1_,*y*_2_] overlaps within the interval [*x*_1_,*x*_2_].
4. When *t*<*s*, *x*_2_>*y*_1_>*x*_1_, and then the left part of the interval [*x*_1_,*x*_2_] intersects the interval [*y*_1_,*y*_2_]; and if *x*_2_>*y*_2_>*x*_1_, then the right part of the interval [*x*_1_,*x*_2_] intersects the interval [*y*_1_,*y*_2_]. Otherwise, if *x*_1_,*x*_2_ is less than *y*_2_ and greater than *y*_1_, then the interval [*x*_1_,*x*_2_] overlaps within the interval [*y*_1_,*y*_2_].

In conclusion, the above scheme can be implemented by means of protocol 1 or 2 and Protocol 3 in this paper.

## Conclusion and discussion

The determination of the relation between a number and a numerical interval is one of the core problems of scientific calculation to protect privacy, and the calculation of the relation between two numbers and numerical intervals on the premise of protecting privacy is also the basic problem of collaborative calculation. At present, most of the solutions reach the integer level while few reach the real number level. This article uses the Bernoulli inequality extension type and combines it with the monotonic function technique to extend the numerical interval to determine the relationship between the real number level, mainly the homomorphic encryption scheme based on two different design size comparisons between the two new real numbers. The Bernoulli inequality extension type reduces the scope of use of the homomorphic encryption system comparison to the range, reduces the encryption algorithm, and improves the efficiency. In addition, a protocol is designed to solve the confidential cooperative determination problem between real numbers by using symbolic functions and other techniques. The protocols in this paper are based on the fact that all parties involved in collaborative computing are semihonest, and the constructed protocols are secure under the semihonest model. The relationship between the number of privacy protections and the numerical interval under the malicious model will be the direction of our future work.
